# Epidemiology and genetic diversity of *Burkholderia pseudomallei* from Riau Province, Indonesia

**DOI:** 10.1371/journal.pntd.0012195

**Published:** 2024-05-28

**Authors:** Dewi Anggraini, Fajri Marindra Siregar, Dani Rosdiana, Rahmat Azhari Kemal, Indra Yovi, Zhana Daisya Triani, Novira Jasmin, Norsila Dwijelita, Jessica R. Webb, Mark Mayo, Mirjam Kaestli, Bart J. Currie

**Affiliations:** 1 Department of Microbiology, Faculty of Medicine, Universitas Riau, Pekanbaru, Indonesia; 2 Arifin Achmad General Hospital, Riau Province, Pekanbaru, Indonesia; 3 Eka Hospital Pekanbaru, Pekanbaru, Indonesia; 4 Department of Biochemistry, Faculty of Medicine, Universitas Riau, Pekanbaru, Indonesia; 5 Department of Internal Medicine, Faculty of Medicine, Universitas Riau, Pekanbaru, Indonesia; 6 Department of Medical Biology, Faculty of Medicine, Universitas Riau, Pekanbaru, Indonesia; 7 Department of Pulmonology, Faculty of Medicine, Universitas Riau, Pekanbaru, Indonesia; 8 Global and Tropical Health Division, Menzies School of Health Research, Charles Darwin University, Darwin, Northern Territory, Australia; 9 Peter Doherty Institute for Infection and Immunity, Melbourne, Victoria, Australia; 10 Department of Infectious Diseases, Royal Darwin Hospital, Darwin, Northern Territory, Australia; University of Florida, UNITED STATES

## Abstract

Melioidosis is a bacterial infection caused by *Burkholderia pseudomallei*, that is common in tropical and subtropical countries including Southeast Asia and Northern Australia. The magnitude of undiagnosed and untreated melioidosis across the country remains unclear. Given its proximity to regions with high infection rates, Riau Province on Sumatera Island is anticipated to have endemic melioidosis. This study reports retrospectively collected data on 68 culture-confirmed melioidosis cases from two hospitals in Riau Province between January 1, 2009, and December 31, 2021, with full clinical data available on 41 cases. We also describe whole genome sequencing and genotypic analysis of six isolates of *B*. *pseudomallei*. The mean age of the melioidosis patients was 49.1 (SD 11.5) years, 85% were male and the most common risk factor was diabetes mellitus (78%). Pulmonary infection was the most common presentation (39%), and overall mortality was 41%. Lung as a focal infection (aOR: 6.43; 95% CI: 1.13–36.59, p = 0.036) and bacteremia (aOR: 15.21; 95% CI: 2.59–89.31, p = 0.003) were significantly associated with death. Multilocus sequence typing analysis conducted on six *B*.*pseudomallei* genomes identified three sequence types (STs), namely novel ST1794 (n = 3), ST46 (n = 2), and ST289 (n = 1). A phylogenetic tree of Riau *B*. *pseudomallei* whole genome sequences with a global dataset of genomes clearly distinguished the genomes of *B*. *pseudomallei* in Indonesia from the ancestral Australian clade and classified them within the Asian clade. This study expands the known presence of *B*. *pseudomallei* within Indonesia and confirms that Indonesian *B*. *pseudomallei* are genetically linked to those in the rest of Southeast Asia. It is anticipated that melioidosis will be found in other locations across Indonesia as laboratory capacities improve and standardized protocols for detecting and confirming suspected cases of melioidosis are more widely implemented.

## Author summary

Melioidosis is a disease caused by the bacterium *Burkholderia pseudomallei*. This disease can affect various organs such as the lungs, skin and soft tissues, central nervous system, internal organs, and others. The clinical manifestations vary, and the lack of laboratory examination support in some countries leads to underreporting of the disease. This also results in a varied death rate, ranging from 10–40% and even higher in some countries. The disease is endemic in the Southeast Asian region and northern Australia and is increasingly recognised in other tropical and sub-tropical locations globally. Indonesia has reported over 100 cases from at least 5 diverse regions. One province in Indonesia, Riau, located on the island of Sumatera bordering Malaysia, has been experiencing a relatively high number of melioidosis cases. This study reports melioidosis cases obtained from two referral hospitals in the province of Riau. The research explores the characteristics of patients, clinical manifestations, and outcomes in these cases. Additionally, the study analyzes factors influencing mortality from melioidosis in Riau. Phylogenetic analysis of six isolates of *B*. *pseudomallei* determined that *B*. *pseudomallei* in the province of Riau, Indonesia is genetically linked to those from neighbouring countries in Southeast Asia.

## Introduction

Melioidosis is an infection caused by *Burkholderia pseudomallei*, a Gram-negative, non-sporing environmental bacterium [1–3]. *B*. *pseudomallei* affects animals and humans and is endemic in Southeast Asia, northern Australia and other tropical and subtropical regions [1–4]. *B*. *pseudomallei* can be transmitted through skin inoculation, inhalation, or ingestion [5–7]. *B*. *pseudomallei* is remarkably resilient and can survive in various hostile environmental conditions, including nutrient deficient soil or even distilled water [8–10]. These bacteria exhibit the ability to persist within host cells by manipulating the host’s immune response, allowing it to evade the immune system [11,12]. *B*. *pseudomallei* is intrinsically resistant to antibiotics used to cover common causes of bacterial sepsis such as first- and second-generation cephalosporins, macrolides, colistin and aminoglycosides [13]. Currently, the management of melioidosis involves two phases: the acute phase, which aims to prevent sepsis-related deaths, and the eradication phase, which focuses on preventing recurrence/ relapse [13–16].

The estimation of melioidosis case numbers is challenging, particularly in regions with limited laboratory diagnostic capacity but also given the wide range of clinical presentations associated with melioidosis [15,17]; however, reports indicate an increasing number of melioidosis cases globally [18,19]. A modelling study conducted in 2016 estimated that there were 165,000 cases of melioidosis worldwide, with a predicted 89,000 deaths annually [20]. South Asian countries accounted for 44% of the predicted burden [20].

In Indonesia, the first case of melioidosis was diagnosed in Cikande, Banten Province in 1929 [21,22]. Similar to other countries in Asia, it is suspected that many cases of melioidosis in Indonesia go undiagnosed or unreported. The modelling study predicted that Indonesia may experience over 20,000 cases of melioidosis annually with over 10,000 deaths [20]. A study analyzing 146 confirmed cases of melioidosis based on culture results from various regions in Indonesia between 2012 and 2017 found an overall mortality rate of 43% [22]. Cases were reported from Sumatera, Java, Kalimantan, Sulawesi and Nusa Tenggara.

Riau Province, situated on Sumatera Island, is predicted to be endemic for melioidosis cases due to its proximity to high-incidence areas like Thailand and Malaysia. Based on the data from the Global Burden of Disease Study 2019, the age-standardized Years of Life Lost (YLL) rates in Riau provinces, attributed to leading causes in 2019, closely align with those of Indonesia [23]. The six predominant risk factors for Disability-Adjusted Life Years (DALYs) in Indonesia for the same year were elevated systolic blood pressure, tobacco consumption, dietary risks, high fasting plasma glucose, increased BMI (body mass index), and child/maternal malnutrition [23]. High BMI emerged as the dominant risk factor for Riau [23]. In this study, we report melioidosis cases observed in two hospitals in Riau Province over the past 13 years, and we also conducted whole genome sequencing on six isolates of *B*. *pseudomallei* from humans to progress insights into the global phylogeography of this species.

## Materials and methods

### Ethics statement

This study received approval from the Ethics Unit of the Faculty of Medicine, Universitas Riau (No. B/120/UN19.5.1.1.8/UEPKK/2021), and informed written consent was obtained from the patients prior to data collection.

### Study locations, subjects and data collection

Riau Province spans an area of 87,023.66 km^2^ and has a population of 6,454,751 individuals. It is situated in the central part of the east coast of Sumatera Island and borders the Malacca Strait, adjacent to Malaysia. The province is traversed by the Siak River. which is the deepest river in Indonesia. The Siak River serves various purposes for the community.

Data for this study were collected from the microbiology laboratories of two hospitals in Riau Province. The data spanned from 1 January 2009, coinciding with the availability of bacterial culture examination in both hospitals, until 31 December 2021. These hospitals are Arifin Achmad Hospital, a government teaching hospital with 598 beds, and Eka Hospital, a private hospital with 300 beds.

*B*. *pseudomallei* identification was conducted using the Vitek 2 System (Biomeriuex, Marcy l’Etoile, France). Each culture of *B*. *pseudomallei* was documented and cross-referenced with the patients’ medical records. Additionally, patient data including age, sex, residence, admission date, clinical risk factors such as diabetes, presence of fever and sepsis, foci of infection, outcomes and type of culture specimens were extracted from the hospital’s record system. Sepsis is defined in accordance with the International Guidelines for the Management of Sepsis and Septic Shock 2021 [24]. Six isolates of *B*. *pseudomallei* obtained from these cases underwent whole genome sequencing and phylogenetic analysis.

### Statistical analysis

In order to asses risk factors or clinical presentations associated with melioidosis mortality, a logistic regression was conducted with outcome death and predictors demographic data, comorbid factors, and clinical presentations (presence of sepsis, fever, lung, and skin and soft tissue as infection foci) of 41 patients with complete data. All variables with p ≤ 0.1 in univariate logistic regression were included in multivariable logistic regression analysis. The final multivariable model used the backward elimination approach, in which variables that had the least significant effect on the model were removed. The crude odds ratio (OR) and adjusted OR (aOR) were calculated during univariate and multivariable modelling, respectively, together with the 95% confidence intervals (95% CIs). All tests were two-sided and P value <0.050 considered statistically significant. All statistical analyses were conducted using SPSS v28.

### Whole genome sequencing of *B*. *pseudomallei* isolates

Genomic DNA was extracted from purified colonies of *B*. *pseudomallei* using the Torax Nucleic Acid Extraction Kit (Torax Biosciences Ltd., Newtownabbey, United Kingdom). The identification of the bacteria was confirmed using a real-time PCR assay that targeted a specific 115-bp segment within the type three secretion system 1 (TTS1) gene of *B*. *pseudomallei* [25]. Whole genome sequencing was performed on an Illumina NovaSeq 6000 platform (Illumina, Inc., San Diego, CA) at the Australian Genome Research Facility (AGRF), generating paired-end reads of 150 bp for each genome. Accession numbers for the six genomes are SAMN37878278 to SAMN37878283 (bioproject PRJNA1029745, SRA SRS19194652 to 7).

Multi-locus sequence types (MLST) were assigned *in silico* using the MLST assignment tool “mlst” (https://github.com/tseemann/mlst) [26] and the PubMLST website (https://pubmlst.org/) [27]. Genomes were assembled *de novo* using shovill 1.1.0 which is based on SPAdes, with minimum length of 1,000 bp and default settings to correct sequencing errors and adaptors (https://github.com/tseemann/shovill) [28]. Average coverage was 128 reads. A core genome alignment was conducted on the six *B*. *pseudomallei* genomes from this study and 121 publicly available, global *B*. *pseudomallei* genomes (S1 Table) resulting in 163,182 core genome single nucleotide polymorphisms (SNPs). This is with using the default settings of Snippy v4.6.0 [29] and the closed *B*. *pseudomallei* genome K96243 as reference (N50 4,074,542 bp; 2 contigs; size 7,247,547 bp, NC_006351.1 and NC_006350.1) [30]. A maximum likelihood phylogenetic tree was generated in IQ-TREE v2.2.0.3 [31] using the nucleotide substitution model TVM+F+I+I+R5 selected by the ModelFinder and lowest BIC score [32] and the final tree was rooted using MSHR668 which was the most ancestral *B*. *pseudomallei* strain in a large phylogenetic study of *B*. *pseudomallei* from across the world and closely related *Burkholderia* species [33]. Bootstrapping was performed using 1,000 replicates (.tre file of consensus tree in S1 File). The tree was visualized using Ggtree v3.6.1 in R v4.1.2 (https://www.r-project.org/) [34]. The sequences of geographical and virulence genetic markers lipopolysaccharide (LPS), *Yersinia*-like fimbrial / *Burkholderia thailandensis*-like flagella and chemotaxis gene cluster (YLF/BTFC), *Burkholderia* intracellular motility A (*bimA)* gene and filamentous hemagglutinin (*fhaB3)* gene were extracted *in silico* using SRST2 v0.2.0 (https://github.com/katholt/srst2/tree/master) [35]: LPS A (*wbil* to *apaH* [location: 3196645–3215231] in K96243 [GenBank ref: NC_006350]), LPS B (*BUC_3392* to *apaH* [location: 499502–535038] in *B*. *pseudomallei* 579 [GenBank ref: NZ_ACCE01000003]), LPS B2 (*BURP840_LPSb01* to *BURP840_LPSb21* [location: 241–26545] in *B*. *pseudomallei* MSHR840 [GenBank ref: GU574442]), BTFC (BURPS668_A0209 in *B*. *pseudomallei* MSHR668 [GenBank ref: CP000571.1]), YLF (*BPSS0124* in *B*. *pseudomallei* K96243 [GenBank ref: NC_006350]), *bimA*_*Bm*_ (*BURPS668_A2118* in *B*. *pseudomallei* MSHR668 [GenBank ref: CP000571.1]), *bimA*_*Bp*_ (*BPSS1492* in *B*. *pseudomallei* K96243 [GenBank ref: NC_006350]) and *fhaB3* (*BPSS2053* in *B*. *pseudomallei* K96243 [GenBank ref: NC_006350]) [36].

## Results

### Demographics, clinical manifestations, risk factors, and outcomes of patients

**Fig 1:**
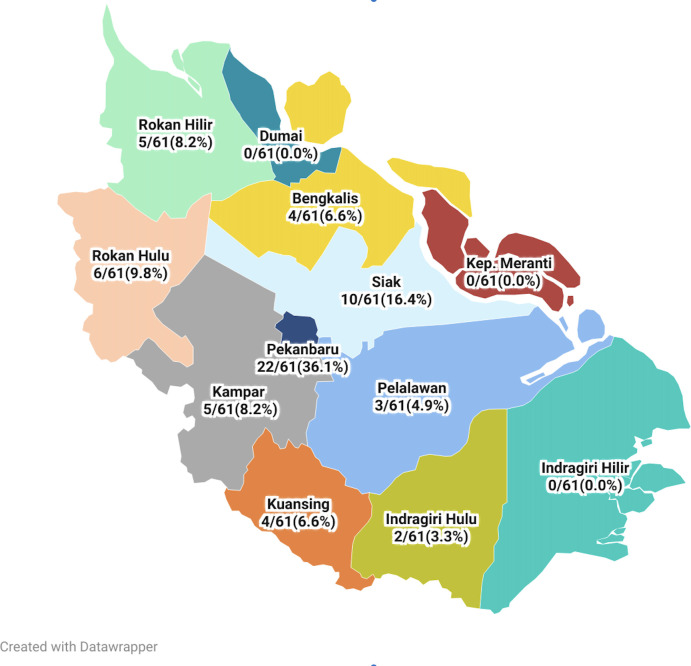
Map of Riau Province and the distribution of patient residences (https://www.datawrapper.de/_/lNhtk/).

Between 2009 and 2021, a total of 68 patients with positive *B*. *pseudomallei* culture results were identified, with 35 patients from Arifin Achmad Hospital and 33 from Eka Hospital Pekanbaru. The patients originated from 9 out of 11 districts in Riau Province, with the commonest being Pekanbaru 26 patients (38.2%), followed by Siak 11 patients (16.2%), Rokan Hulu 7 patients (10.3%), Rokan Hilir 4 patients (5.9%) and others 20 patients (29.4%) (Fig 1).

Due to retrospective data collection and one of the hospitals not utilizing electronic medical records, complete data for all variables were only obtained for 41 out of 68 patients. Subsequent data analysis was conducted on the 41 patients with complete data. Table 1 describes the demographic characteristics, risk factors, clinical manifestations, patient outcomes, and the source of patient culture specimens. Among the patients with data available, the majority were male (85%), and 28% of patients were between 41–60 years of age (mean age 49.1) (SD 11.5) (Table 1). The distribution of cases was similar between the dry (49%) and rainy (51%) seasons. The most commonly identified risk factor was diabetes mellitus, affecting 32/41 patients (78%). Fever was reported in 27/41 cases (66%), while sepsis was documented in 22/41 cases (51%). The most frequently observed focal infection was in the lungs, affecting 16/41 cases (39%). The mortality rate from melioidosis was 41% (17/41 cases).

**Table 1:** Demographics, risk factors, clinical manifestations and patient outcomes.

Variables	Complete sample
	(n = 41)
**Demographics**	
**Male**	35 (85%)
**Age, years (mean [SD])**	49.1 (11.5)
**Age, years**	
0–10	0 (0%)
11–20	2 (5%)
21–30	0 (0%)
31–40	5 (12%)
41–50	14 (34%)
51–60	14 (34%)
61–70	6 (15%)
71–80	0 (0%)
**Hospital admission**	
Dry season (March-August)	20 (49%)
Rainy Season (September-February)	21 (51%)
**Risk factors**	
Diabetes mellitus	32 (78%)
Human immunodeficiency virus (HIV) infection	0 (0%)
Hazardous alcohol use	1 (2%)
Chronic kidney disease	10 (24%)
Chronic lung disease	2 (5%)
**Clinical manifestation**	
**Primary focus of infection**	
Lungs	16 (39%)
Skin and soft tissue	8 (20%)
No evidence focal infection	7 (17%)
Bone and joint	2 (5%)
Lymph nodes	3 (7%)
Central nervous system	2 (5%)
Ear	1 (2%)
Dental	1 (2%)
Pericardial	1 (2%)
**Fever**	27 (66%)
**Sepsis**	21 (51%)
**Bacteremia**	21 (51%)
**Outcome**	
Recovered	24 (59%)
Death from melioidosis	17 (41%)
**Specimen origin of *B*.*pseudomallei* isolate**	
Blood	21 (51%)
Pus	13 (32%)
Sputum	7 (17%)

Univariate analysis found that sepsis (OR: 4.00; 95% CI: 1.06–15.14, p = 0.037), lung as a primary focus of infection (OR: 4.29; 95% CI: 1.13–16.31, p = 0.029), and bacteremia (OR: 11.33; 95% CI: 2.46–52.15, p<0.001) were associated with death (Table 2). In a multivariable model, lung as a focal infection (aOR: 6.43; 95% CI: 1.13–36.59, p = 0.036) and bacteremia (aOR: 15.21; 95% CI: 2.59–89.31, p = 0.003) were significantly associated with death (Table 2).

**Table 2:** *Risk factors for mortality of melioidosis*.

Variable	Outcome		Univariate analysis		Multivariable analysis	
	Death (n = 17) n (%)	Recovered (n = 24) n (%)	OR (95% CI)	P-value	aOR (95% CI)	P-value
Sex Male Female	15 (88%) 2 (12%)	20 (83%) 4 (17%)	1.50 (0.24–9.30)	1.000		
Age > = 50 years	9 (53%)	13 (54%)	0.95 (0.27–3.31)	0.938		
Diabetes mellitus	14 (82%)	18 (75%)	1.56 (0.33–7.34)	0.711		
Chronic kidney disease	4 (24%)	6 (25%)	0.92 (0.22–3.95)	1.000		
Fever	11 (65%)	16 (67%)	0.92 (0.25–3.39)	0.896		
Sepsis	12 (71%)	9 (38%)	4.00 (1.06–15.14)	0.037*		
Lung as primary focus of infection	10 (59%)	6 (25%)	4.29 (1.13–16.31)	0.029*	6.43 (1.13–36.59)	0.036*
Skin and soft tissue as primary focus of infection	2 (12%)	6 (25%)	0.40 (0.07–2.28)	0.433		
Bacteremia	14 (82.4%)	7 (29.2%)	11.33 (2.46–52.15)	<0.001*	15.21 (2.59–89.31)	0.003*

*significance at p = 0.05

OR: odds ratio; aOR: adjusted odds ratio; CI: confidence interval

### Bacterial genomics and phylogenetic analysis

There were 6 isolates that were retrieved and available for WGS examination, originating from 5 patients. Isolates R6 and R9 originated from the same patient, with an interval between the two episodes of 4 months, and the patient did not receive oral eradication therapy, suggesting the likelihood of a relapse infection [37] (Table 3). MLST analysis revealed three STs, consisting of ST1794 (n = 3), ST46 (n = 2), and ST289 (n = 1). ST1794 is a novel isolate and has to date only been detected in Indonesia. Two isolates of ST1794 originated from the patient with relapsed melioidosis, and this patient and the other with ST1794 both came from the same district, Siak. All six genomes contained the YLF, LPS type B and *Bp bimA* markers. With the exception of R2, all isolates also contained the *fhaB3* gene (Table 4).

**Table 3:** *B*. *pseudomallei* strains included in this study: Patient and clinical presentation details.

Isolate	Patient	Hospital	Age (year)	Sex	Isolate date	Outcome	Primary focus of infection	Sepsis	Specimen	Resident
R2	1	EH	48	Male	14 Nov 2019	Recovered	Skin and soft tissue	Yes	Blood	Pekanbaru
R3	2	EH	52	Female	26 Dec 2019	Recovered	CNS	No	Pus subdural	Siak
R6	3	EH	62	Male	19 May 2020	Recovered	Lung	Yes	Sputum	Siak
R9	3	EH	62	Male	4 Jan 2020	Recovered	Lung	No	Sputum	Siak
R10	4	AA	50	Male	30 Nov 2020	Death	Skin and soft tissue	No	Pus chest wall	Rokan Hilir
R11	5	EH	40	Male	16 Feb 2021	Recovered	Lung	Yes	Blood	Siak

**Table 4:** *B*. *pseudomallei* strains included in this study: Geographical and genetic markers.

Isolate	MLST	YLF-BTFC	LPS	*bimA*	*fhaB3*
R2	46	YLF	A	*Bp*	Negative
R3	1794	YLF	A	*Bp*	Positive
R6	1794	YLF	A	*Bp*	Positive
R9	1794	YLF	A	*Bp*	Positive
R10	46	YLF	A	*Bp*	Positive
R11	289	YLF	A	*Bp*	Positive

**Fig 2:**
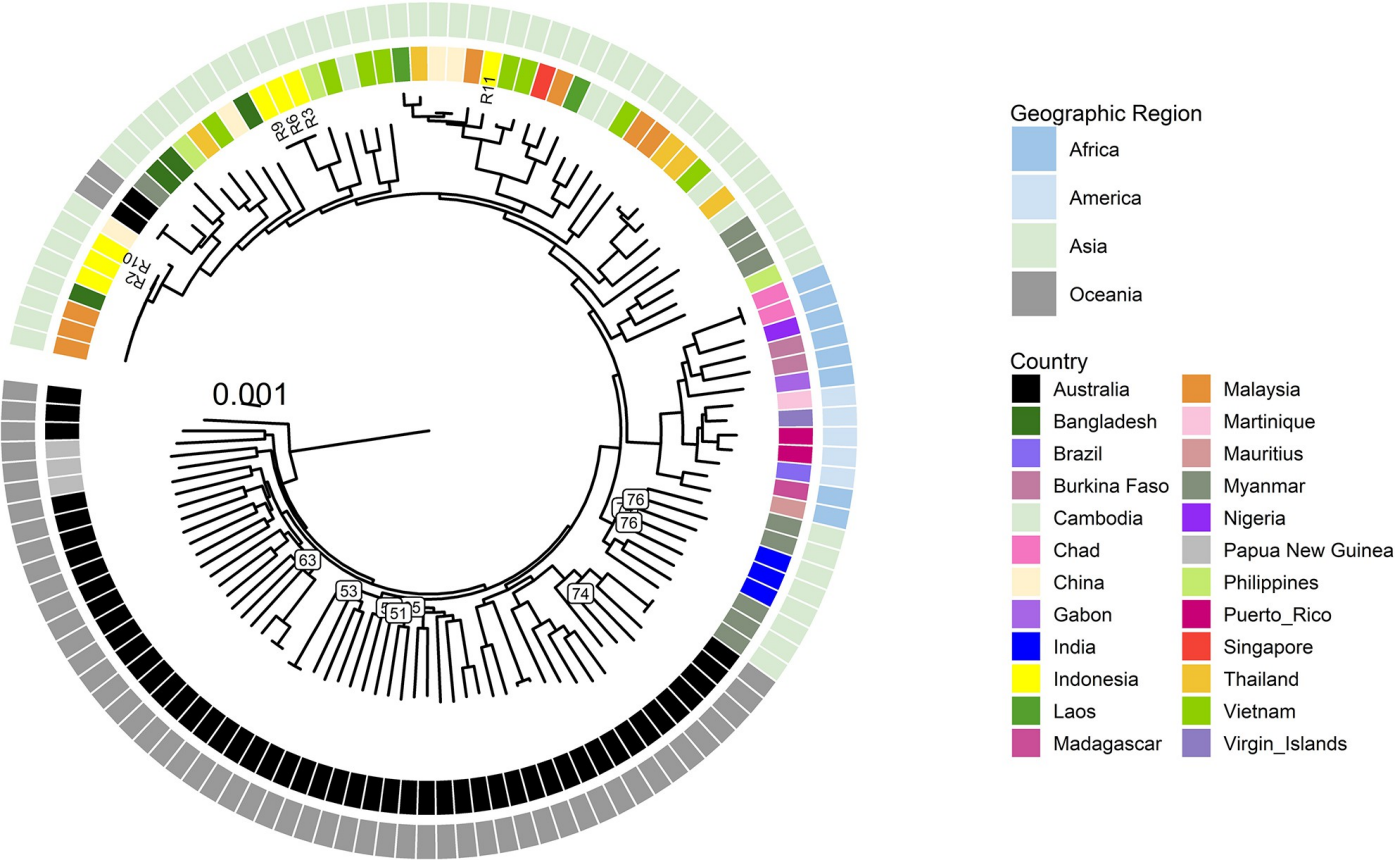
Maximum likelihood phylogenetic tree of *B*. *pseudomallei* from Riau (n = 6) with a global set of genomes (n = 121) and 163,182 core genome SNPs. The scale bar indicates substitutions per site. Bootstrap support (using 1,000 replicates) for branches below 80% are shown revealing some deeper nodes with lower support while all main nodes were well supported.

The phylogenetic analysis showed a clear clustering of the Indonesian *B*. *pseudomallei* genomes with *B*. *pseudomallei* genomes from other Asian countries (Fig 2). ST46 isolates R2 and R10 were closely related with each other (21 core SNPs difference) and other ST46 isolates from Malaysia and Bangladesh. R6 and R9 from the same patient had no core SNPs, confirming this was a relapsed infection. There was a difference of 12 SNPs between R3 and R6, both from patients with ST 1794 *B*. *pseudomallei* who lived in the same district. In contrast, there were 13,560 and 12,180 SNPs’ difference between the Indonesian isolates from different nodes and with different STs, such as R9 compared to R11 or R9 compared to R2. In comparison, there was a difference of 17,200 SNPs between R2 and an African isolate and 17,890 SNPs between R2 and an Australian *B*. *pseudomallei* genome.

## Discussion

To the best of the authors’ knowledge, this study is the first to report epidemiological and clinical data on melioidosis from a specific region in Indonesia and to include bacterial genomic analysis. Our findings showed that the demographic characteristics, including gender and age, of melioidosis patients in this region of Indonesia, are similar to previous studies conducted in Indonesia and other countries such as Thailand, Singapore, Malaysia, China, and Australia. [22,38–42].

In Riau Province, the frequency of melioidosis cases during the dry and rainy seasons was nearly equal. This contrasts with northern Australia where the rainy season is a strong independent risk factor for melioidosis, with cases linked to rainfall in the two weeks before the onset of symptoms [43]. In Hainan, China, melioidosis cases peaked in August and September, coinciding with the rainy season, with the peak year being the one with the highest rainfall [41]. *B*. *pseudomallei* has been found in both, shallow and deeper soil levels [6]. During the rainy season these bacteria can proliferate and move to the surface, possibly due to aerotaxis [44,45]. The rainy season can therefore lead to increased exposure of individuals to muddy soil and water containing *B*. *pseudomallei* [44,45]. Data from the Riau Province Central Statistics Office indicates that there is a consistent pattern of both moderate and high rainfall throughout the entire year, in contrast to the Northern Territory of Australia which has a “wet-dry tropics” climate. Further investigation is necessary to examine the correlation between melioidosis occurrences and the fluctuating rainfall patterns in the Riau province, both within the year and between years.

The most common risk factor identified in this study was diabetes mellitus (DM) (78%). Approximately 80% of melioidosis patients have risk factors, including DM, hazardous alcohol consumption, and chronic lung disease [30,46]. In previous studies conducted in Indonesia, DM was the most prevalent risk factor, albeit with a lower percentage of 36% [22]. DM is also a major risk factor for melioidosis in Malaysia, China, and Australia [40–42]. DM impairs the immune system by suppressing chemotaxis, phagocytosis, and cytokine response to bacterial killing, thereby increasing the risk of infection [47]. Polymorphonuclear leukocytes (PMN) are critical components of the innate immune response against *B*. *pseudomallei* [47]. However, the PMN response and the release of chemokines for IL-8 activation are delayed in DM [47]. Considering that Indonesia ranks fifth globally in terms of the number of DM cases, with 19.5 million estimated patients in 2021, proper identification and comprehensive management of melioidosis cases are crucial [48].

The most frequently reported clinical symptom among the patients in this study was fever (66%). In Singapore, fever was present in 81% of melioidosis cases (39). The most common presenting focus of infection observed was the lungs (39%). In previous research in Indonesia, the lungs were found to be the primary focus in 25% of cases [22]. These results from Indonesia are lower rates of pneumonia than seen in studies from Malaysia, Australia, and China, where pneumonia accounted for 41%, 52%, and 54% of presentations, respectively [40–42]. The lower rates of pneumonia from Indonesia may reflect ascertainment issues in data collection from the relatively small number of cases and further prospective studies are now required in Indonesia. The potential for aerosol spread of *B*. *pseudomallei* during the rainy season, possibly caused by rainfall during strong winds, may contribute to pneumonia as the most common symptom [44]. In this study, sepsis was recorded for 51% cases. In Kelantan, Malaysia, hospitalized patients with melioidosis had sepsis 69%, severe sepsis in 26%, and septic shock in 34% [40]. In the Darwin prospective melioidosis study 21% melioidosis cases had strictly defined septic shock [42].

The melioidosis mortality rate in Riau Province, according to this study, was 41% which is similar to reported 43% in a previous study in Indonesia [22], higher than reported from Malaysia (33%) and China (23%) [40,41]. Melioidosis death rates have declined from over 50% to under 10% in some other regions of the world [42]. However, numerous areas where melioidosis is prevalent still lack access to essential laboratory and treatment facilities, with severe resource limitations [15]. In this study, sepsis, lung as focus of infection and bacteremia were identified as risk factors for mortality due to melioidosis. This was consistent with other studies, showing that sepsis [49] and bacteremia [50] are strongly linked to mortality in melioidosis.

When we commenced this study, there was only a single record of *B*. *pseudomallei* from humans in Indonesia available in the international Multilocus Sequence Typing (MLST) database (https://pubmlst.org/bpseudomallei). WGS analysis showed a large genetic variability of the Indonesian genomes suggesting that *B*. *pseudomallei* has been established in Riau province for a long time. ST 46 is the most common isolate in Southeast Asia, with more than 150 records on the MLST website and was seen in three of our patients. ST289 is also seen in other Southeast Asian countries and was found in one patient in our study. We found a new MSLT type 1794 in three different patients. WGS also showed that all Riau province isolates contained the geographical marker LPS type A which is the most common LPS type in Thailand and Australia [51], as well as the YLF cluster which is widespread in Southeast Asia [52]. R2 was the only *fhaB3* negative isolate and of interest, R2 was from a patient with cutaneous melioidosis. The absence of *fhaB3* has been associated with localized skin lesions [53]. All isolates also contained the *Bp bimA* gene allele which is the more abundant allele in Southeast Asia [53]. The alternate *bimA* gene allele, *Bm bimA*, has been associated with neurological disease [54].

There are several limitations that should be acknowledged in this study. Firstly, the identification of *B*. *pseudomallei* for the majority of isolates was conducted using a phenotypic approach without genotypic confirmation. Only six isolates in this study were genetically confirmed as *B*. *pseudomallei*. The second limitation pertains to the completeness of the clinical data. Although efforts were made to collect comprehensive clinical information, there were remaining data gaps in this retrospective study.

## Conclusion

The data collected in this study provides a valuable snapshot of the clinical presentation and mortality rate of melioidosis in a specific area of Indonesia. This disease is more commonly observed in males, typically impacting middle-aged individuals, with diabetes mellitus being the predominant risk factor. The lungs are the commonest site of infection. The mortality rate is notably high, comparable to that in some other developing nations. The WGS analysis effectively differentiates the genomes of *B*. *pseudomallei* in Indonesia from the ancestral Australian *B*. *pseudomallei*, clearly placing Indonesian *B*. *pseudomallei* within the Asian *B*. *pseudomallei* clade. It is expected that melioidosis may exist in many other regions of Indonesia, and this potential will be unmasked with the enhancement of laboratory capabilities and the widespread adoption of standardized guidelines for investigating suspected melioidosis cases. Further studies and WGS of larger and more geographically dispersed collections of *B*. *pseudomallei* are now needed to expand the understanding of the epidemiology of melioidosis and the phylogeography of *B*. *pseudomallei* across Indonesia.

## Supporting information

S1 Table Isolates in Phylogenetic Tree. (PDF)

S2 Table Dataset Melioidosis Patients. (XLSX)

S1 File Consensus Maximum Likelihood Phylogenetic Tree File -.tre file can be opened with FigTree. (TRE)
