# Supplementary material for: Epidemiology and genetic diversity of Burkholderia pseudomallei from Riau Province, Indonesia
Source: PLoS Negl Trop Dis. 2024 May 28;18(5):e0012195. doi: 10.1371/journal.pntd.0012195 (PMC11161056; doi:10.1371/journal.pntd.0012195)
Supplement: S1 Table — (PDF) [file pntd.0012195.s001.pdf]

**Table S1: Isolates in Phylogenetic Tree**

| Isolate | Year | MLST | Geographical Area | Country of Origin | Accession |
|---|---|---|---|---|---|
| MSHR 11369 [1] | 2017 | 36 | Australia Papua New Guinea | Australia | SAMN12824619 |
| MSHR 10743 [1] | 2017 | 109 | Australia Papua New Guinea | Australia | SAMN12824552 |
| MSHR 10130 [2] | | 113 | Australia Papua New Guinea | Australia | SRR9333951 |
| MSHR 10167 [1] | 2017 | 131 | Australia Papua New Guinea | Australia | SAMN12824497 |
| MSHR 10751 [1] | 2017 | 132 | Australia Papua New Guinea | Australia | SAMN12824554 |
| MSHR 10246 [1] | 2017 | 144 | Australia Papua New Guinea | Australia | SAMN12824504 |
| MSHR 1555 [3] | 2003 | 239 | Australia Papua New Guinea | Australia | ERR539745 |
| MSHR 11042 | 2017 | 279 | Australia Papua New Guinea | Australia | SAMN12824580 |

| | | | | | |
|---|---|---|---|---|---|
| [1] | | | | | |
| MSHR 10166 [1] | 2017 | 320 | Australia Papua New Guinea | Australia | SAMN12824496 |
| MSHR 6137 [4] | | 325 | Australia Papua New Guinea | Australia | AXDS00000000 |
| MSHR 10888 [1] | 2017 | 326 | Australia Papua New Guinea | Australia | SAMN12824566 |
| MSHR 10117 [1] | 2017 | 327 | Australia Papua New Guinea | Australia | SAMN12824493 |
| MSHR 0091 [5] | | 331 | Australia Papua New Guinea | Australia | SRR8790694 |
| MSHR 10690 [1] | 2017 | 335 | Australia Papua New Guinea | Australia | SAMN12824540 |
| MSHR 11384 [1] | 2017 | 362 | Australia Papua New Guinea | Australia | SAMN12824620 |
| MSHR 9671 [1] | 2016 | 456 | Australia Papua New Guinea | Australia | SAMN12824487 |

| | | | | | |
|---|---|---|---|---|---|
| MSHR 11277 [1] | 2017 | 462 | Australia Papua New Guinea | Australia | SAMN12824609 |
| MSHR 10901 [1] | 2017 | 464 | Australia Papua New Guinea | Australia | SAMN12824567 |
| MSHR 10622 [1] | 2017 | 466 | Australia Papua New Guinea | Australia | SAMN12824531 |
| MSHR 10100 [1] | 2017 | 472 | Australia Papua New Guinea | Australia | SAMN12824490 |
| MSHR 10269 [1] | 2017 | 553 | Australia Papua New Guinea | Australia | SAMN12824507 |
| MSHR 5848 [6] | | 553 | Australia Papua New Guinea | Australia | CP008909, CP008910 |
| MSHR 11290 [1] | 2017 | 561 | Australia Papua New Guinea | Australia | SAMN12824611 |
| MSHR 10541 [1] | 2017 | 562 | Australia Papua New Guinea | Australia | SAMN12824528 |
| MSHR 10978 | 2017 | 566 | Australia Papua New Guinea | Australia | SAMN12824574 |

| | | | | | |
|---|---|---|---|---|---|
| [1] | | | | | |
| MSHR 10697 [1] | 2017 | 616 | Australia Papua New Guinea | Australia | SAMN12824542 |
| MSHR 11267 [1] | 2017 | 639 | Australia Papua New Guinea | Australia | SAMN12824607 |
| MSHR 10283 [2] | | 678 | Australia Papua New Guinea | Australia | SRR9333956 |
| MSHR 10274 [2] | | 731 | Australia Papua New Guinea | Australia | SRR9333954 |
| MSHR 9932 [5] | | 734 | Australia Papua New Guinea | Australia | SRR8790686 |
| MSHR 0938 [5] | | 737 | Australia Papua New Guinea | Australia | SRR8790683 |
| MSHR 2254 [5] | | 770 | Australia Papua New Guinea | Australia | SRR8790690 |
| MSHR 11194 | 2017 | 801 | Australia Papua New Guinea | Australia | SAMN12824596 |

| | | | | | |
|---|---|---|---|---|---|
| [1] | | | | | |
| MSHR 11424 [5] | | 807 | Australia Papua New Guinea | Australia | SRR8790701 |
| MSHR 10550 [1] | 2017 | 809 | Australia Papua New Guinea | Australia | SAMN12824529 |
| MSHR 10693 [1] | 2017 | 813 | Australia Papua New Guinea | Australia | SAMN12824541 |
| MSHR 10746 [1] | 2017 | 982 | Australia Papua New Guinea | Australia | SAMN12824553 |
| MSHR 10526 [1] | 2017 | 984 | Australia Papua New Guinea | Australia | SAMN12824525 |
| MSHR 4378 [7] | | 1025 | Australia Papua New Guinea | Australia | JQDP00000000 |
| MSHR 7343 [7] | | 1030 | Australia Papua New Guinea | Australia | JQDM00000000 |
| MSHR 2618 [5] | | 1485 | Australia Papua New Guinea | Australia | SRR8790693 |

| | | | | | |
|---|---|---|---|---|---|
| MSHR 10126 [2] | | 1591 | Australia Papua New Guinea | Australia | SRR9333952 |
| MSHR 10275 [2] | | 1651 | Australia Papua New Guinea | Australia | SRR9333953 |
| MSHR 10259 [1] | 2017 | 1654 | Australia Papua New Guinea | Australia | SAMN12824506 |
| MSHR 11266 [1] | 2017 | 1655 | Australia Papua New Guinea | Australia | SAMN12824606 |
| MSHR 10355 [1] | 2017 | 1656 | Australia Papua New Guinea | Australia | SAMN12824512 |
| MSHR 11335 [1] | 2017 | 1658 | Australia Papua New Guinea | Australia | SAMN12824615 |
| MSHR 11255 [1] | 2017 | 1659 | Australia Papua New Guinea | Australia | SAMN12824604 |
| MSHR 11092 [1] | 2017 | 1660 | Australia Papua New Guinea | Australia | SAMN12824589 |

| | | | | | |
|---|---|---|---|---|---|
| MSHR 10619 [1] | 2017 | 1704 | Australia Papua New Guinea | Australia | SAMN12824530 |
| MSHR 10635 [1] | 2017 | 1705 | Australia Papua New Guinea | Australia | SAMN12824533 |
| MSHR 668 [9] | 1995 | 129 | Australia Papua New Guinea | Australia | SRR1617372 |
| 6_CDC [3] | 1960 | 46 | Asia | Bangladesh | ERR311033 |
| H061220286 [3] | 2006 | 1007 | Asia | Bangladesh | ERR298751 |
| H034580128 [3] | 2003 | unknown | Asia | Bangladesh | ERR298750 |
| H103520155 [3] | 2010 | unknown | Asia | Bangladesh | ERR298752 |
| H061740680 [3] | 2006 | 92 | Americas | Brazil | ERR298754 |
| MSHR 7964 [8] | | 1121 | Africa | Burkina Faso | SRR3145394 |

| | | | | | |
|---|---|---|---|---|---|
| MSHR 7965 [8] | | 1122 | Africa | Burkina Faso | SRR3145395 |
| E562 [3] | 2006 | 494 | Asia | Cambodia | ERR178253 |
| E569 [3] | 2006 | 510 | Asia | Cambodia | ERR178254 |
| SR_025 [3] | 2007 | 690 | Asia | Cambodia | ERR178251 |
| SR_020 [3] | 2007 | 693 | Asia | Cambodia | ERR178250 |
| SR_039 [3] | 2008 | 694 | Asia | Cambodia | ERR178245 |
| 5691 [3] | 1956 | 82 | Africa | Chad | ERR298779 |
| MSHR 6969 [3] | 1956 | 82 | Africa | Chad | ERR298347 |
| Hainan_106 [3] | 1996 | 50 | Asia | China | ERR298758 |
| E0702 [3] | 1999 | 70 | Asia | China | ERR539771 |
| HK2 [3] | 1987 | 70 | Asia | China | ERR539766 |
| Hainan1 [3] | 1996 | unknown | Asia | China | ERR298757 |

| | | | | | |
|---|---|---|---|---|---|
| Gabon_Bp [3] | 2013 | 1127 | Africa | Gabon | ERR403716 |
| G9709 [3] | 1995 | 43 | Asia | India | ERR311037 |
| H054640145 [3] | 2005 | 344 | Asia | India | ERR298759 |
| H103360117 [3] | 2010 | unknown | Asia | India | ERR298760 |
| MSHR2056 [3] | 2005 | 46 | Asia | Indonesia | ERR539754 |
| MM37 [3] | 2004 | 500 | Asia | Laos | ERR162603 |
| MM39 [3] | 2004 | 511 | Asia | Laos | ERR162598 |
| MSHR7966 [8] | | 1043 | Africa | Madagascar | SRR3145396 |
| LN_10 [3] | NA | 46 | Asia | Malaysia | ERR298768 |
| LN_31348 [3] | NA | 46 | Asia | Malaysia | ERR298765 |
| LN_6 [3] | NA | 46 | Asia | Malaysia | ERR298766 |

| | | | | | |
|---|---|---|---|---|---|
| LN_34170 [3] | 1977 | 51 | Asia | Malaysia | ERR298763 |
| MSHR0315 [3] | 1994 | 232 | Asia | Malaysia | ERR311047 |
| LN_29564 [3] | NA | 289 | Asia | Malaysia | ERR298764 |
| LN_22892 [3] | NA | 438 | Asia | Malaysia | ERR298767 |
| MSHR7400 [3] | 2010 | 92 | Americas | Martinique | ERR298359 |
| Green [3] | 2004 | unknown | Africa | Mauritius | ERR311038 |
| MSHR12627 [10] | 2017 | 56 | Asia | Myanmar | SAMN15594681 |
| MSHR12629 [10] | 2018 | 90 | Asia | Myanmar | SAMN15594683 |
| MSHR12634 [10] | 2018 | 346 | Asia | Myanmar | SAMN15594687 |
| MSHR12633 [10] | 2018 | 1371 | Asia | Myanmar | SAMN15594686 |

| | | | | | |
|---|---|---|---|---|---|
| MSHR 12636 [10] | 2018 | 1752 | Asia | Myanmar | SAMN15594688 |
| MSHR 12637 [10] | 2018 | 1753 | Asia | Myanmar | SAMN15594689 |
| MSHR 12833 [10] | 2018 | 1765 | Asia | Myanmar | SAMN15594694 |
| MSHR 12837 [10] | 2018 | 1766 | Asia | Myanmar | SAMN15594695 |
| MSHR 12647 [10] | 2018 | 1770 | Asia | Myanmar | SAMN15594693 |
| H101180656 [3] | 2010 | 707 | Africa | Nigeria | ERR298772 |
| G1467 [3] | 1978 | 246 | Australia Papua New Guinea | Papua New Guinea | ERR311036 |
| MSHR 0141 [3] | 1992 | 274 | Australia Papua New Guinea | Papua New Guinea | ERR311041 |

| | | | | | |
|---|---|---|---|---|---|
| MSHR 2434 [3] | 2006 | 515 | Australia Papua New Guinea | Papua New Guinea | ERR539760 |
| MK_441 [3] | 1990 | 57 | Asia | Philippines | ERR298773 |
| MK_451 [3] | 1990 | 98 | Asia | Philippines | ERR298774 |
| H110440887 [3] | 2011 | unknown | Asia | Philippines | ERR298777 |
| H0929 [3] | 1998 | 92 | Americas | Puerto_Rico | ERR311039 |
| F3253 [3] | 1982 | 95 | Americas | Puerto_Rico | ERR311035 |
| MSHR 6975 [3] | 1935 | 51 | Asia | Singapore | ERR298353 |
| 2769a [3] | 2001 | 33 | Asia | Thailand | ERR162621 |
| 2698a [3] | 2001 | 34 | Asia | Thailand | ERR162620 |
| 2659a [3] | 2001 | 54 | Asia | Thailand | ERR162614 |
| E361 [3] | 1998 | 230 | Asia | Thailand | ERR162626 |

| | | | | | |
|---|---|---|---|---|---|
| 137 [3] | 2008 | 67 | Asia | Vietnam | ERR178256 |
| 720 [3] | 2009 | 67 | Asia | Vietnam | ERR178260 |
| 532 [3] | 2009 | 163 | Asia | Vietnam | ERR178258 |
| MSHR 6973 [3] | 1953 | 288 | Asia | Vietnam | ERR298351 |
| 1097 [3] | 2010 | 367 | Asia | Vietnam | ERR178268 |
| 683 [3] | 2009 | 871 | Asia | Vietnam | ERR178259 |
| 1040 [3] | 2010 | 884 | Asia | Vietnam | ERR178261 |
| 1042 [3] | 2010 | unknown | Asia | Vietnam | ERR178262 |
| H065460522 [3] | 2007 | unknown | Americas | Virgin_Islands | ERR298778 |
| R2 [This Study] | 2019 | 46 | Asia | Indonesia | SAMN37878278 |
| R3 [This Study] | 2019 | 1794 | Asia | Indonesia | SAMN37878279 |
| R6 [This Study] | 2020 | 1794 | Asia | Indonesia | SAMN37878280 |

| | | | | | |
|---|---|---|---|---|---|
| R9 [This Study] | 2020 | 1794 | Asia | Indonesia | SAMN37878281 |
| R10 [This Study] | 2020 | 46 | Asia | Indonesia | SAMN37878282 |
| R11 [This Study] | 2021 | 289 | Asia | Indonesia | SAMN37878283 |

# Reference


1. Rachlin A, Mayo M, Webb JR, Kleinecke M, Rigas V, Harrington G, et al. Whole-genome sequencing of *Burkholderia pseudomallei* from an urban melioidosis hot spot reveals a fine-scale population structure and localised spatial clustering in the environment. Sci Rep. 2020 Mar 25;10(1):5443.

2. Kaestli M, O'Donnell M, Rose A, Webb JR, Mayo M, Currie BJ, et al. Opportunistic pathogens and large microbial diversity detected in source-to-distribution drinking water of three remote communities in Northern Australia. PLoS Negl Trop Dis. 2019;13(9):e0007672.

3. Chewapreecha C, Holden MT, Vehkala M, Välimäki N, Yang Z, Harris SR, et al. Global and regional dissemination and evolution of *Burkholderia pseudomallei*. Nat Microbiol. 2017 Jan 23;2:16263.

4. McRobb E, Sarovich DS, Price EP, Kaestli M, Mayo M, Keim P, et al. Tracing melioidosis back to the source: Using whole-genome sequencing to investigate an outbreak originating from a contaminated domestic water supply. J Clin Microbiol. 2015 Apr;53(4):1144-8.

5. Webb JR, Rachlin A, Rigas V, Sarovich DS, Price EP, Kaestli M, et al. Tracing the environmental footprint of the *Burkholderia pseudomallei* lipopolysaccharide genotypes in the tropical "Top End" of the Northern Territory, Australia. PLoS Negl Trop Dis. 2019; 13(7): e0007369.

6. Daligault HE, Davenport KW, Minogue TD, Bishop-Lilly KA, Broomall SM, Bruce DC, et al. Whole-genome assemblies of 56 *Burkholderia* species. Genome Announc. 2014 Nov 20;2(6):e01106-14.

7. Johnson SL, Baker AL, Chain PS, Currie BJ, Daligault HE, Davenport KW, et al. Whole-Genome Sequences of 80 Environmental and Clinical Isolates of *Burkholderia pseudomallei*. Genome Announc. 2015 Feb 12;3(1):e01282-14.

8. Sarovich DS, Garin B, De Smet B, Kaestli M, Mayo M, Vandamme P, et al. Phylogenomic analysis reveals an Asian origin for African *Burkholderia pseudomallei* and further supports

melioidosis endemicity in Africa. mSphere. 2016;1(2):e00089-15.

9. Johnson SL, Bishop-Lilly KA, Ladner JT, Daligault HE, Davenport KW, Jaissle J, et al. Complete genome sequences for 59 Burkholderia isolates, both pathogenic and near neighbor. Genome Announc. 2015 Apr 30;3(2):e00159-15.

10. Webb JR, Win MM, Zin KN, Win KKN, Wah TT, Ashley EA, *et al.* Myanmar *Burkholderia pseudomallei* strains are genetically diverse and originate from Asia with phylogenetic evidence of reintroductions from neighbouring countries. Sci Rep. 2020;10:16260.
